# Single-cell RNA binding protein regulatory network analyses reveal oncogenic HNRNPK-MYC signalling pathway in cancer

**DOI:** 10.1038/s42003-023-04457-2

**Published:** 2023-01-21

**Authors:** Weiwei Zhou, Qiuling Jie, Tao Pan, Jingyi Shi, Tiantongfei Jiang, Ya Zhang, Na Ding, Juan Xu, Yanlin Ma, Yongsheng Li

**Affiliations:** 1 College of Bioinformatics Science and Technology, Harbin Medical University, Harbin, Heilongjiang 150081 China; 2 Hainan Provincial Key Laboratory for Human Reproductive Medicine and Genetic Research, Hainan Clinical Research Center for Thalassemia, Reproductive Medical Center, National Center for International Research “China-Myanmar Joint Research Center for Prevention and Treatment of Regional Major Disease”, The First Affiliated Hospital of Hainan Medical University, Hainan Medical University, Haikou, 571199 China; 3 College of Biomedical Information and Engineering, Hainan Women and Children’s Medical Center, Hainan Medical University, Haikou, 571199 China

**Keywords:** Computational biology and bioinformatics, Systems biology, Tumour immunology

## Abstract

RNA-binding proteins (RBPs) are key players of gene expression and perturbations of RBP-RNA regulatory network have been observed in various cancer types. Here, we propose a computational method, RBPreg, to identify the RBP regulators by integration of single cell RNA-Seq (N = 233,591) and RBP binding data. Pan-cancer analyses suggest that RBP regulators exhibit cancer and cell specificity and perturbations of RBP regulatory network are involved in cancer hallmark-related functions. We prioritize an oncogenic RBP-HNRNPK, which is highly expressed in tumors and associated with poor prognosis of patients. Functional assays performed in cancer cells reveal that HNRNPK promotes cancer cell proliferation, migration, and invasion in vitro and in vivo. Mechanistic investigations further demonstrate that HNRNPK promotes tumorigenesis and progression by directly binding to MYC and perturbed the MYC targets pathway in lung cancer. Our results provide a valuable resource for characterizing RBP regulatory networks in cancer, yielding potential biomarkers for precision medicine.

## Abstract (web summary)

A computational pipeline and webserver identify regulators of RNA-binding proteins (RBPs) at single-cell resolution and are applied to determine the role of RBP HNRNPK in cancer.

## Introduction

RNA-binding proteins (RBPs) are key players of gene expression in post-transcriptional events^1^ and perturbation of RBP-RNA regulatory network has been observed in various cancer types^2–4^. Recent advances of cross-linking and immunoprecipitation (CLIP) have provided exciting opportunities to map transcriptome-wide binding sites of RNA-binding proteins^5,6^. However, there is still a lack of computational methods to comprehensively identify the critical RBP regulators in cancer.

The transcriptional state of a cell is strictly regulated by numbers of transcription factors (TFs) and RBPs. Considering the important roles of RBPs, perturbation of their RNA-binding function can impact many downstream genes and pathways, leading to complex diseases phenotypes^7^. Transcriptome-wide analyses have revealed numerous RBPs perturbed in various cancer types. For example, the RBP SERBP1 was found to function as an oncogenic factor in glioblastoma by bridging cancer metabolism and epigenetic regulation^8^. RBP FXR1 has been found to drive cMYC translation by recruiting eIF4F complex to the translation start site in cancer^9^. Integrated analysis of multidimensional data had revealed EIF2S2 can promote tumorigenesis and progression by regulating MYC-mediated inhibition via FHIT-related enhancers in gastrointestinal cancer^10^. These results suggested that the transcriptome analysis provided comprehensive insights into the function of RBPs.

Moreover, the development of single-cell sequencing technologies has led new biological insights into regulation of gene expressions^11^. A few methods have been proposed to infer the cell types from gene expression (i.e., SingleR^12^ and CaSTLe^13^) and predict the interactions between TFs and target genes (i.e., SECNIC^14^). Yet the dynamics of RBP regulation in single cells is largely unknown. STAMP (Surveying Targets by APOBEC-Mediated Profiling) was developed to detect RBP-RNA interactions in single cells^15^. However, it is still difficult to determine the RBP activities in single cells, as well as prioritize the critical RBP regulators in cancer.

In this study, we proposed a computational method RBPreg, which was based on RBP to gene expression associations (GENIE3^16^) that were filtered for genes containing the respective RBP binding motif identified with MEME^17^, to identify the RBP regulators by integration of single-cell RNA-Seq (scRNA-Seq) and RBP binding data. We demonstrated that RBPreg can be exploited to identify the critical regulators in cell types of interest and the RBP regulators exhibited cell type and cancer specificity. In particular, we prioritized an oncogenic RBP-HNRNPK, which potentially interacts with MYC to promote cancer cell proliferation, migration, and invasion. This study provided a generally application method to identify RBP regulators and shed lights into the mechanisms of RBP regulation in cancer.

## Results

### Overview of RBPreg: a computational pipeline for identification of RBP regulators in cancer

**Fig. 1:**
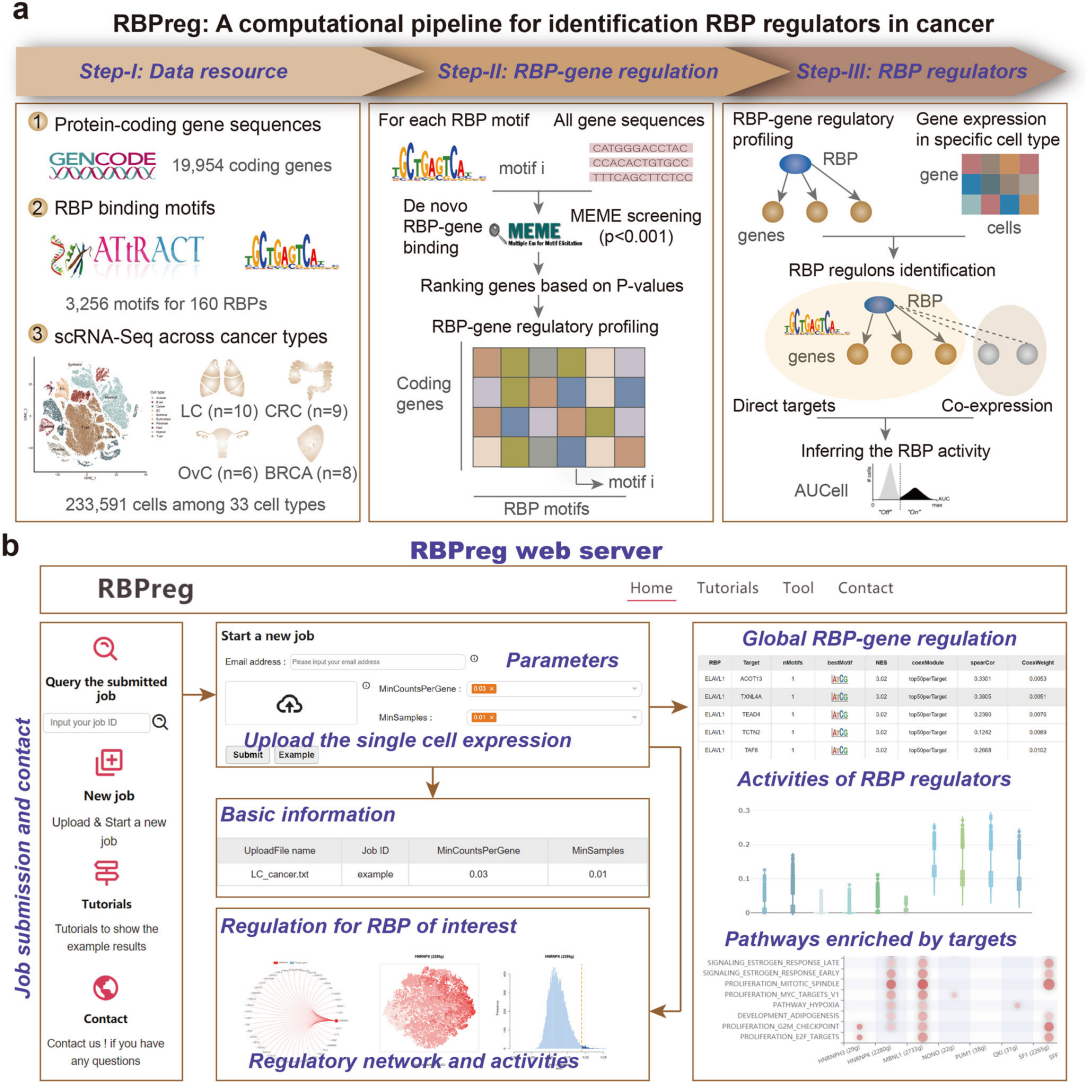
The RBPreg pipeline for identifying RBP regulators by integrating scRNA-Seq and regulation data. **a** The workflow of RBPreg computational pipeline. **b** Illustration of the usage of RBPreg web server and the results showing RBP-gene regulation in cancer.

RBPs are critical regulators of gene expression and play fundamental roles in cancer^9,10^. However, there is still lack of computational method to identify the functionally important RBPs^18^, particular based on single-cell sequencing data. Here, we proposed an integrated computational pipeline to identify RBP regulators in cancer specific cell types. This pipeline integrated the RBP binding motifs, genomic sequences of genes and single cell-based gene expression (Fig. 1a). Motivated by the idea of SECNIC, this method was method based on RBP to gene expression associations (identified by GENIE3^16^) that are filtered for genes containing the respective RBP binding motif identified with MEME. We first *de novo* scanned the gene and identified the RBP motifs in gene sequences. We found that ~80% genes on average were considered to have RBP binding sites. Moreover, for each RBP motif, we calculated the proportion of binding sites observed in introns or exons. We found that there were 81.36% of RBP motifs were more likely observed in introns of the genes (Supplementary Data 1). All protein coding genes were ranked based on the significance levels. Next, the RBP-gene regulatory correlations were evaluated based on the expression correlation between RBP and gene (identified by GENIE3^16^). Finally, the activities of RBPs in specific cell types were evaluated by AUCell.

Moreover, we proposed a user-friendly web server for conveniently using the RBPreg pipeline. Users can upload the customized scRNA-Seq gene expression profiles and identify the activated RBP regulators in corresponding cell types (Fig. 1b). The results will be returned to the email address provided by the users when submitting their jobs. They can also retrieve the results for previously submitted job by the unique job IDs. By submitting the single-cell gene expression, RBPreg provides user-friendly functionalities, ultra-efficient calculation, intuitive table and figure visualization interface to display the RBP-gene regulation, activities of RBP regulators and pathways enriched by targets (Fig. 1b). Additional filtering options and elaborate application notebooks were provided in this webserver (Fig. S1). We anticipated that RBPreg pipeline will provide an opportunity for better understanding the regulatory mechanisms of RBPs in human complex diseases.

### RBP regulators exhibit cancer and cell specificity

**Fig. 2:**
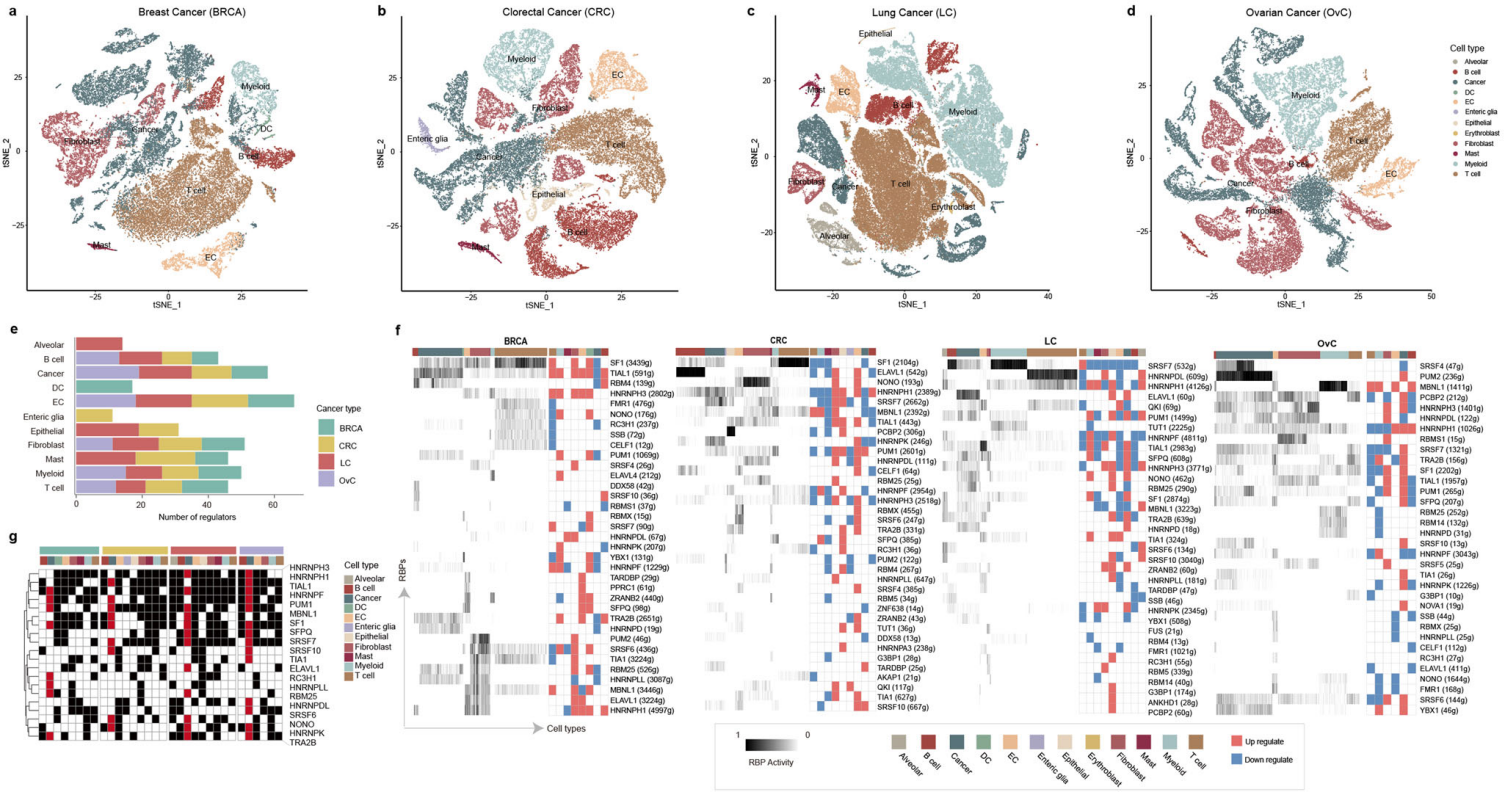
RBP regulators identified in four cancer types. **a**–**d** t-SNE representation for BRCA, CRC, LC and OvC. Color-coded for cell types. **e** The number of RBP regulators identified in different cell types across cancers. **f** Heatmap of RBP regulatory activity in different cell types across cancers. The numbers with the parenthesis mean the number of potentially target genes of RBPs. The heat maps on the right panels were for differential expressions of RBPs. Red for upregulated and blue for downregulated. **g** Heatmap showing the commonly activated RBP regulators in pan-cancer. The black and red in the heap map indicated that the RBPs were active in corresponding cell types and red is highlighting the cancer cell column.

Genes exhibited tissue or cell type specific expression^19,20^, which are regulated by number of transcription factors and RBPs^21,22^. Based on the expressions of marker genes (Supplementary Data 2), we classified the cells into different cell types (Fig. 2a–d). In total, we obtained 44,024 cells of 8 cell types in breast cancer (BRCA), 44,684 cells of 9 cell types in colorectal cancer (CRC), 93,575 cells of 10 cell types in lung cancer (LC), and 45,114 cells of 6 cell types in ovarian cancer (OvC) (Supplementary Data 3). By analysis of the top highly expressed genes in each cluster, we found that the classical marker genes exhibited high expression in corresponding cell types (Fig. S2-S3 and Supplementary Data 2). For example, CD3E and CD2 were highly expressed in T cells, CD79A was highly expressed in B cells and KRT18 was highly expressed in cancer cells (Fig. S3).

To evaluate the performance of RBPreg, we applied it to the single-cell transcriptome of four cancers. In total, we identified 100 RBP regulators in BRCA, 114 in CRC, 131 in LC and 88 in OvC (Supplementary Data 4). We also identified several RBP motifs that were significantly enriched in general (Supplementary Data 5). For example, there were three motifs (UCCCCCAA_1029, ACCCCCCCCCUA_s61 and CCCCCCC_1026) of HNRNPK were significantly enriched in lung cancer (Fig. S4). Moreover, we found that the majority of target genes for RBPs were significantly supported by public eCLIP-Seq data (Fig. S5), although the Jaccard index was lower since the comparisons are between different cell contexts (Supplementary Data 6). We found that the gene expressions of different cell types were regulated by diverse numbers of RBP regulators (Fig. 2e). In particular, we identified 43 and 46 RBP regulators in B cells and T cells, respectively. There were 49 activated RBPs identified in four cancer types. Moreover, we found that the RBP regulators exhibited distinct activities across cell types (Fig. 2f). PUM2 exhibited higher activity in ovarian cancer cells (Fig. 2f), and targeting PUM2 has been identified as an effective way to reverse cisplatin resistance in OvC^23^. HNRNPDL exhibited higher activity in T cells of lung cancer (Fig. 2f), and it has been demonstrated that RNPL regulates T cell differentiation and migration by regulating pre-T cell receptor and chemokine receptor signaling^24^. These results suggested that RBPreg can identify the RBP regulators that play important roles in corresponding cell types.

Moreover, we compared the RBP regulators across cancer types (Fig. S6). In total, 20 RBPs were identified in all cancers (Fig. 2g), which was significantly larger than those of random conditions (*p* < 0.001, random test). Several RBPs exhibited widespread activities in various cell types and cancer types, such as HNRNPH3, HNRNPH1 and TIAL1 (Fig. 2g and Fig. S7). However, we also found that these RBPs exhibited activities in different cell types. For example, ELAVL1 exhibited specific activity in lung cancer cells (Fig. 2g), which has been found to play critical roles in lung cancer^25,26^. HNRNPK exhibited higher activities in CRC, LC and OvC cancer cells but not in BRCA (Fig. 2g). Emerging evidence has indicated the critical roles of HNRNPK in regulating a wide range of biological processes and disease pathogenesis^27–29^. Moreover, we compared the RBP-gene regulatory networks across cancer types. We found that the RBP regulatory networks were statistically similar in the same cell types across cancer types (Fig. S8). Taken together, RBP regulators exhibit cancer and cell specific activities and play a fundamental role in various cancer types.

### RBPreg prioritizes oncogenic HNRNPK in lung cancer

**Fig. 3:**
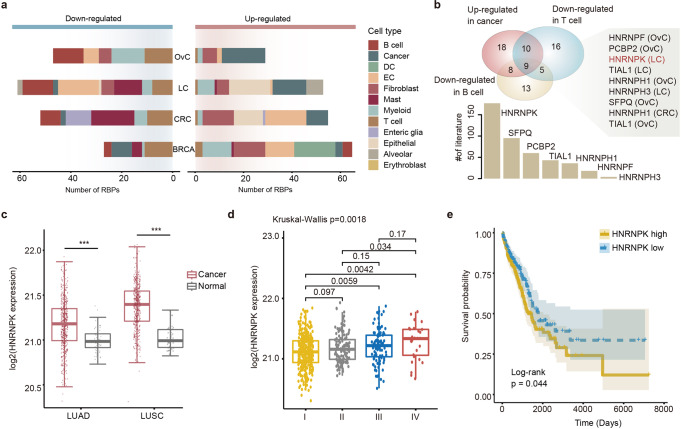
Prioritization of oncogenic RBP regulator HNRNPK in lung cancer. **a** Numbers of differentially expressed RBP regulators in different cell types across cancers. Left for downregulated and right for upregulated RBP regulators. **b** The top panel showing the overlap of RBPs upregulated in cancer and downregulated in immune cells. The comparison was performed by cancer type. The aberrations of cancer types indicated in brackets were the cancers in which the RBP regulator was identified. The bottom bar plots showing the number of literatures for RBP regulators. **c** Boxplots showing the expressions of HNRNPK in LUAD and LUSC. ***p < 0.001 for Wilcoxon’s rank sum tests. Error bars showing the first and third quantiles. **d** Boxplots showing the expressions of lung cancer patients with different stages. Error bars showing the first and third quantiles. Comparison among groups was by Kruskal–Wallis test and between two groups by Wilcoxon’s rank sum tests. **e** Kaplan–Meier survival analysis of lung cancer patients (LUAD) stratified by the median expression levels of HNRNPK.

Prioritizing the genes or regulators that act as drivers of cancer remains a crucial bottleneck in cancer therapeutic development^30,31^. We thus investigated the prioritized RBP regulators by RBPreg in detail. First, we analysed the expression of RBP regulators across cell types. We found that numbers of RBPs exhibited specifically high or low expression across cell types (Fig. 2f and Supplementary Data 7). There were higher numbers of RBPs exhibiting higher expression in cancer cells of BRCA (3), CRC (9), LC (14) and OvC (18) (Fig. 3a), when comparing with other immune cell types. In particular, we identified seven RBPs exhibiting higher expression in cancer cells but lower expression in T and B cells in corresponding cancer type (Fig. 3b). Emerging evidence has suggested that these RBPs play fundamental roles in cancer and literature mining revealed there were 4 to 177 papers reporting their associations with cancer (Fig. 3b and Supplementary Data 8). It has been unveiled that SFPQ plays a central role in regulating alternative splicing and response to platinum in OvC^32^. HNRNPH1 is frequently upregulated in multiple cancer cells and involved in tumorigenesis^33^.

In particular, HNRNPK was reported to be associated with cancer development in 177 papers (Fig. 3b). We also found that HNRNPK exhibited significantly higher expression in lung cancer cells than other immune cells (Fig. S9). Moreover, we investigated the expression of HNRNPK in lung cancer two cohorts from TCGA project. We found that HNRNPK was highly expressed in cancer patients of lung adenocarcinoma (LUAD) and lung squamous cell carcinoma (LUSC) (Fig. 3c, *p*-values < 0.001 for Wilcoxon’s rank sum test). Moreover, patients in high stages were with significantly higher expression of HNRNPK in lung cancer (Fig. 3d, *p* = 0.0018 for Kruskal–Wallis test). We also explored the association of HNRNPK expression with patient survival. The patients were classified into high-risk and low-risk based on the median expression of HNRNPK. We found that patients with higher expression of HNRPK exhibited poor survival in lung cancer but not in other cancer types (Fig. 3e and Fig. S10, log-rank *p* = 0.044). All these results suggested that HNRNPK functions as an oncogene and play important roles in lung cancer.

### HNRNPK promotes tumor growth and invasion in vitro and in vivo

**Fig. 4:**
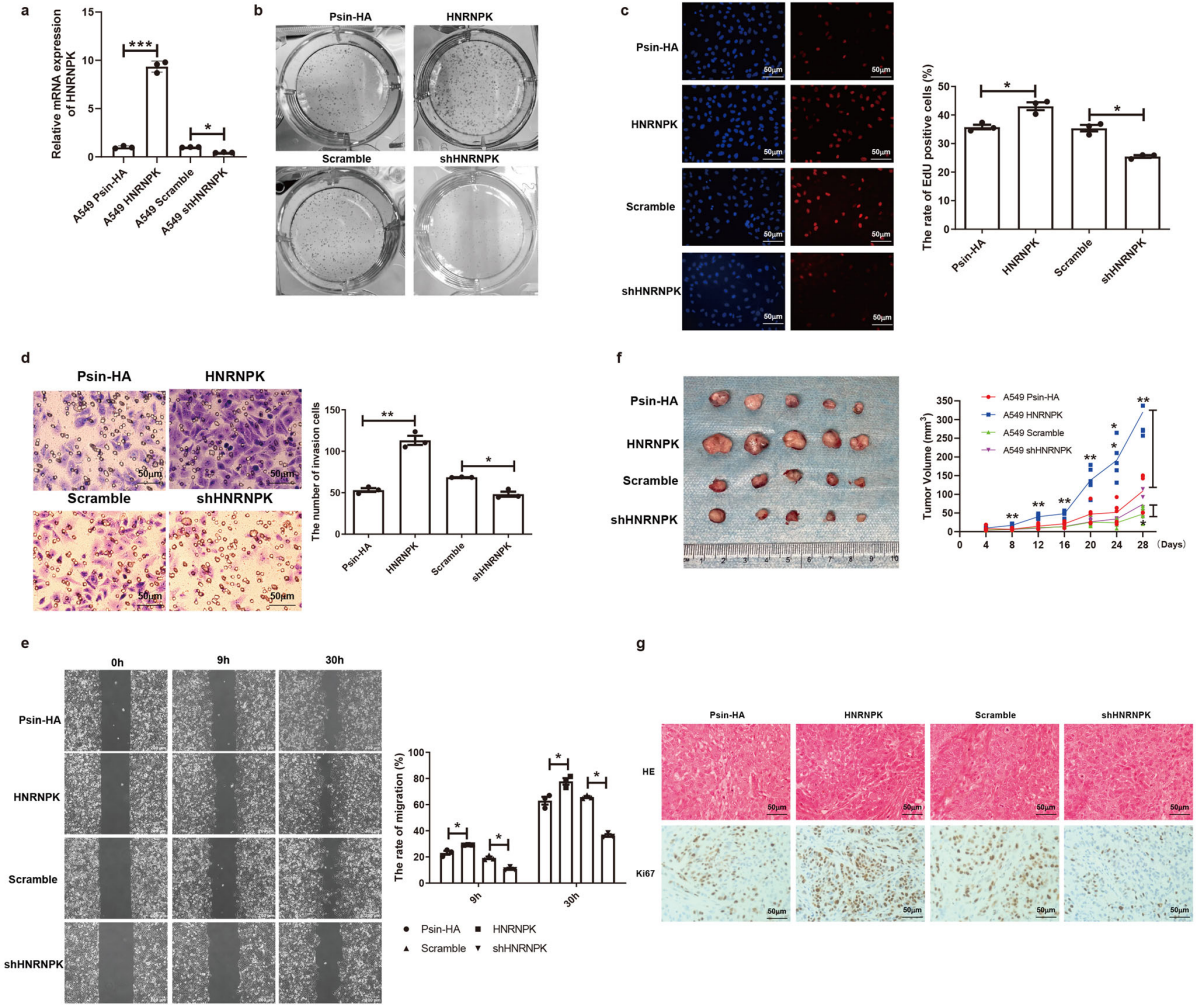
HNRNPK promote cancer cell growth, invasion and migration. **a** Relative mRNA expressions of HNRNPK in overexpression and knockdown conditions. Error bars showing the variances. **b** Colony formation assays of the effects of overexpression/knockdown of HNRNPK vs. controls. **c** The rate of EdU positive cells for overexpression/knockdown of HNRNPK vs. controls. Error bars showing the variances. **d** The number of invasion cells for overexpression/knockdown of HNRNPK vs. controls. Error bars showing the variances. **e** The rate of migration of cancer cells for overexpression/knockdown of HNRNPK vs. controls. Error bars showing the variances. **f** Tumor volumes for overexpression/knockdown of HNRNPK vs. controls. **g** IHC for Ki67 in overexpression/knockdown of HNRNPK vs. controls.

To further validate the molecular functions of HNRNPK, we performed a series of functional assay in cell line and mouse models. We first constructed a shRNA and overexpression HNRNPK lentiviral vectors to construct knockdown and overexpression HNRNPK cell models separately to explore the biological function of HNRNPK in human lung cancer cell line A549 cells. The effects of HNRNPK knockdown and overexpression on the mRNA expression of HNRNPK was verified by Real-time PCR (Fig. 4a), which showed that the expression of HNRNPK mRNA levels were decreased or increased in HNRNPK knockdown or overexpressed A549 cells, respectively. These results showed that the stable HNRNPK knockdown (shHNRNPK) and overexpression (HNRNPK) A549 cells were successfully generated. We next evaluated the effects of HNRNPK expression on the cell proliferation by EdU and colony formation assays. The results showed that HNRNPK knockdown suppressed A549 cell proliferation (Fig. 4b, c). Conversely, overexpressed HNRNPK remarkably promoted cell proliferation (Fig. 4b, c). These findings suggested that HNRNPK promoted A549 cells proliferation.

To investigate the effects of HNRNPK on A549 cells migration and invasion, we next performed wound-healing and transwell assays on HNRNPK knockdown and overexpression cells, respectively. The transwell assay revealed that knockdown HNRNPK inhibited, but overexpressed HNRNPK enhanced cell invasion (Fig. 4d). In addition, the wound-healing results showed that HNRNPK knockdown significantly inhibited the A549 cell migration while overexpressed HNRNPK promoted cell migration (Fig. 4e). These results indicate that HNRNPK promotes the migration and invasion in A549 cells in vitro.

To further confirm the function of HNRNPK in A549 cells in vivo, we constructed a mouse model of human lung cancer xenograft. Knockdown and overexpression HNRNPK cell as well as their control cells were delivered into nude mice, and tumor growth was monitored and compared. We evaluated the tumor progression by comparing the tumor size, growth curve, and tumor weight among different groups. The results indicated that overexpression HNRNPK promoted tumorigenesis, as reflected by increased tumor size and tumor weight in the model. Meanwhile, the knockdown of HNRNPK significantly impaired tumor progression, as reflected by decreased tumor size and tumor weight in the model (Fig. 4f). Ki67 has become a very important indicator to evaluate the activity of tumor cells^34,35^. Next, the expression of HNRNPK and Ki67 were measured by IHC. We found that the expression of HNRNPK and Ki67 were increased in mice delivered overexpression HNRNPK cells, while the expression of HNRNPK and Ki67 were significantly decreased in mice delivered knockdown HNRNPK cells (Fig. 4g). Together, these results demonstrated that HNRNPK promotes tumor growth, invasion and migration in vitro and in vivo.

### HNRNPK perturbs MYC singling pathways in cancer

**Fig. 5:**
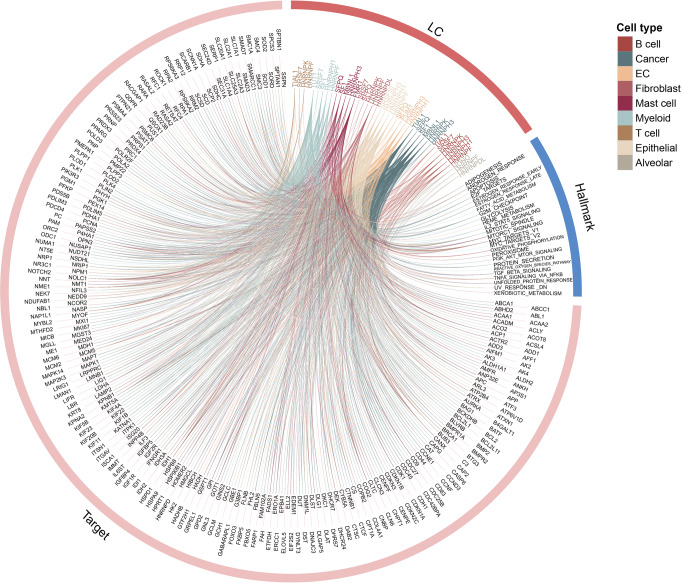
Circos plot for functions of RBP regulators in lung cancer. RBPs and genes were linked if the genes were potential targets of corresponding RBPs. Genes annotated to cancer hallmarks were linked to corresponding pathways. Lines were colored by cancer types. The colors of RBP gene names indicated in which cell type the RBP was identified.

To gain insights into the potential targeting pathways of RBP regulators, we next performed functional enrichment analysis based on the target genes. We found that the targets of numerous RBPs were significantly enriched in cancer hallmark-related pathways (Fig. 5 for lung cancer, Fig. S11 and Supplementary Data 9–10). In addition, we found that these RBPs exhibited higher activities in various cell types. We next calculated the number of RBP regulators for each pathway and found that several pathways were regulated by >10 RBPs (Fig. S12). Mitotic spindle and MYC targets were regulated by more RBPs across all four cancers. Proper organization of the mitotic spindle is key to genetic stability^36^ and the MYC oncogene and its targets contribute to the genesis of many human cancers^37^. These results suggested that RBPreg identified number of RBP regulators that play important roles in cancer.

**Fig. 6:**
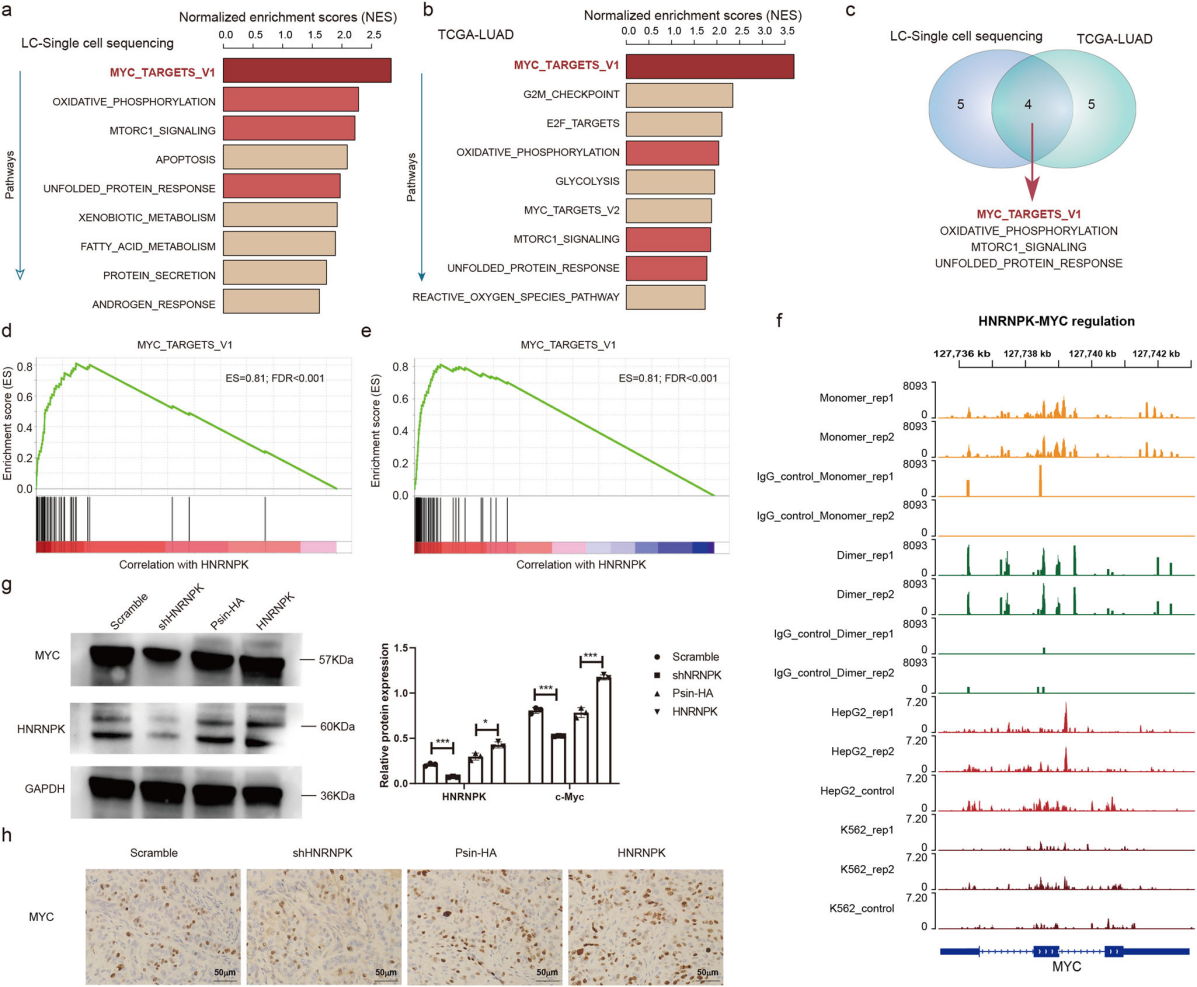
HNRNPK-MYC signaling pathway perturbation in lung cancer. **a**, **b** Pathways enriched by potential targets of HNRNPK. a was based on single cell RNA-seq data and b was for TCGA bulk transcriptome analysis. **c** Venn plot showing the overlap of pathways in single-cell and bulk transcriptomes. **d**, **e** Enrichment plots of MYC target pathway identified by genes coexpressed with HNRNPK in lung cancer. **d** for single cell transcriptome analysis and e for bulk transcriptome analysis. **f** Genome visualization of HNRNPK binding with MYC in cell lines. **g** Relative protein expression of MYC in overexpression or knockdown of HNRNPK lung cancer cells. Error bars represent the standard errors. **h** IHC of MYC in overexpression or knockdown of HNRNPK lung cancer cells.

We next performed GSEA analysis based on the target genes to further investigate the functional pathways of HNRNPK. Based on the single-cell and bulk transcriptome of lung cancer, we identified 9 cancer hallmark-related pathways that were potentially activated by HNRNPK (Fig. 6a, b and Fig. S13). Moreover, four pathways (MYC targets, oxidative phosphorylation, MTORC1 signaling, and unfolded protein response) were identified in two datasets (Fig. 6c). Leading edge gene analysis identified 38 and 34 genes involving MYC targets pathway in single-cell and bulk transcriptome, respectively (Fig. 6d, e). In total, 31 genes were overlapped in both datasets and 12 genes (i.e., CNBP, HDAC2, LDAH, RPL6 and EIF4G2) were annotated in Cancer Gene Census or CancerMine^38,39^. We next downloaded the public CLIP-seq and eCLIP-seq of HNRNPK and investigated the read distributions around MYC. Importantly, our CLIP-seq and eCLIP-seq analyses revealed a strong association between HNRNPK and the MYC. The presence of HNRNPK binding sites on the MYC transcript were observed in cell lines (Fig. 6f). There were clear peaks in HNRNPK CLIP-seq data but not observed in IgG and control datasets. We also found that there were several binding sites of HNRNPK in MYC mRNAs in the FIMO scanning. However, when we integrated the single cell sequencing data in lung cancer, we found that MYC was not observed in potential target gene list of HNRNPK. These results suggested that context-specific regulation of RBPs in cancer.

To further assess the effect of HNRNPK in regulating the translation of MYC mRNA, we next detected the expression of MYC in knockdown and overexpression HNRNPK cells and mice model. We observed that HNRNPK knockdown resulted in a decrease in MYC expression in A549 cells and mice model (Fig. 6g, h). Conversely, HNRNPK overexpression resulted in an increase in MYC expression in A549 cells and mice model (Fig. 6g, h), indicating that HNRNPK may regulate MYC levels. All these results suggested that HNRNPK plays important functions in lung cancer by perturbing the MYC signaling pathway.

## Discussion

Rapid progresses in high throughput sequencing technologies have identified numerous of RBPs and perturbations in RBP-gene regulatory network have been associated with cancer development^1,3^. A few methods have been proposed to infer the co-expression networks or identify the TFs regulators from single-cell RNA-seq data, but there is still no method to predict the RBP regulators in cancer. In this study, we proposed the computational pipeline-RBPreg to prioritize the RBP regulators in distinct cell types by integration of single-cell transcriptome and RBP regulation in cancer. In addition, a web server was set up for facilitating the identification of RBP regulators. The framework proposed in this study can be used to analyse other cancer types and we expected that RBPreg will be extremely useful for understanding the function of RBPs in cancer.

We next applied RBPreg to public single cell RNA-seq data across four cancer types and identified 100 RBP regulators in BRCA, 114 in CRC, 131 in LC and 88 in OvC. Numbers of papers reported their associations with cancer (Fig. 3b and Supplementary Data 8). We also evaluated the associations between expressions of all RBP regulators and overall survival of patients. We found that several RBPs were associated with overall survival across cancer types (Fig. S14 and Supplementary Data 11). To further validate the RBP-gene regulation predicted by computational method, we analysed the public eCLIP-seq and shRNA-seq data across cancer cell lines. We found that the majority of RBP-gene regulations were supported by experimental data, suggesting the accuracy of the predicted regulations. Moreover, we used the same strategy in SCENIC and selected the targets of RBPs with most significant *p*-value. A transcript with multiple good binding sites might be more likely to be regulated by an RBP. However, the most likely targets of RBPs are still predicted based on computational methods. As the eCLIP-Seq data increase, we will update the RBPreg method in the future by integrating the more confident RBP regulation data.

Moreover, we found that the RBP regulators exhibited high cell type specificity and cancer specificity. We built the XGBoost classifiers based on the expression of RBP regulators, and found that the classifiers can accurately distinguish corresponding cell types from other cells. The AUCs ranged from 0.670 to 0.986 (Fig. S15). In addition, we further evaluated whether the RBP regulons could be used to distinguish cancer cells from different cancer types. We found that the classifiers constructed based on the expressions of RBP regulons reached 0.71–0.76 in four cancer types (Fig. S16). Tissue specificity is an important aspect of many genetic diseases and various types of regulators, such as TFs, long non-coding RNAs and microRNAs, have shown extensive tissue specific expression patterns in cancer^19,40^. These results are an important step towards the comprehensive characterization of RBP functions in specific cell types of cancers. Based on functional enrichment analysis, we found that the prioritized RBP regulators were mainly involved in cancer-related pathways. In particular, we prioritized the HNRNPK in lung cancer and validated its functions in cell lines and animal models.

Many studies have identified HNRNPK as an oncogene, and it is central to many cellular events, such as lncRNA regulation (i.e., Neat1, Lncenc1 and Xist), activation of p53/p21 pathways and bone homeostasis^27^. In this study, we revealed the association between HNRNPK and MYC signaling pathway in lung cancer. Although emerging studies have revealed the important functions of HNRNPK and MYC in cancer, limited evidence were supported in lung cancer. In this study, we found that there were HNRNPK binding motifs in MYC mRNAs. But when we integrated the single cell RNA sequencing data to identify the potentially regulators in lung cancer, we found that MYC was not a strong candidate target. The CLIP-seq data greatly supported the RBP-gene regulation identified in this study, suggesting that the genes identified in the HNRNPK regulatory network directly post-transcriptionally regulated by HNRNPK (Fig. S5). We also found that HNRNPK has binding sites around MYC in three cell lines. In addition, protein-protein interaction data from STRING suggested that HNRNPK physically interacts with MYC. We also found that HNRNPK might co-regulate downstream target genes with MYC (Fig. S17), and play important roles in cancer. Together, all these observations suggest that HNRNPK might perform its complex functions in multiple ways in different cancer contexts. DNA-methylation-induced silencing of DIO3OS has been demonstrated to drive non-small cell lung cancer progression via activating HNRNPK-MYC-CDC25A axis^41^. We demonstrated that HNRNPK functions as an oncogene in lung cancer by binding MYC mRNAs to promote the cancer development and progression. These results make HNRNPK as a valuable pre-clinical candidate for assessment of novel therapeutics in lung cancer. However, we found that HNRNPK shows common essentiality across all cancer cell lines in the DepMap^42^. Targeting pan-essential genes may lead to broad cytotoxic effects^43^, which may limit the application of HNRNPK as pre-clinical candidate. Thus, additional functional experiments in cancer preclinical models will be needed to make efforts to benefit cancer patients.

In summary, our study proposed a computational pipeline and developed a webserver to identify the RBP regulators at single-cell resolution. Our applications of RBPreg in cancers provided a valuable resource for characterizing RBP regulatory networks, and reveal oncogenic HNRNPK-MYC signaling pathway in lung cancer.

## Methods

### Cell culture

A549 cell line was donated by Key Laboratory of Emergency and Trauma, Ministry of Education, Hainan Medical University. 293 T cell line was purchased from Institute of Cell Biology (Shanghai, China). Both cells were cultured in DMEM (Gibco, Invitrogen, Carlsbad, CA, USA) containing 10% fetal bovine serum (FBS) (Gibco, Invitrogen, Carlsbad, CA, USA) supplemented with 1% penicillin and streptomycin.

### Cell transfection

Plasmids for HNRNPK knockdown (shRNA) and HNRNPK overexpression were purchased from Genechem Co., LTD (Shanghai, China) and empty vector-transfected cells were established as a control. 293 T cells were transfected with plasmids encoding HNRNPK shRNA or HNRNPK overexpression, as well as packaging plasmids (pSPAX2 and pMD2.G) using the calcium phosphate method. Culture supernatant was collected 48 h and 72 h after transfection, respectively. Before transfection, A549 cells were incubated overnight and then separately transfected with an appropriate volume of virus solution. Transfected cells were screened using 2 μg/ml puromycin (ST551, Beyotime, China) for 7 days. Then, HNRNPK expression was verified by Real-time PCR. A549 cells with HNRNPK knockdown were obtained and designated as A549-shHNRNPK. A549 cells with HNRNPK overexpression were obtained and designated as A549-HNRNPK.

### RNA isolation, real-time PCR

Total RNA was extracted using TRIzol reagent (Life technology, USA) according to the manufacturer’s instruction. The RNA concentration was measured and complementary DNA was synthesized using reverse transcriptase kit (Takara, Dalian, China) according to the instruction. Real-time PCR was carried out using SYBR Premix Ex Taq^TMII^ (Takara, Dalian, China) with Mx3000p QPCR system (Agilent, CA, USA). The primers sequences were showed in Supplementary Data 12.

### Western blot

Protein samples were extracted using RIPA buffer (89900, Fisher, USA) containing protease inhibitor (S8830, Sigma, USA). Protein concentration was measured using a BCA reagent kit (P0012, Beyotime, China). A total of 20 µg cell protein was separated by SDS-PAGE, and then transferred to PVDF membranes. After blocking, the membranes were incubated with the anti-HNRNPK (1:2000 dilution, ab52600, Abcam, UK), anti-MYC (1:1000 dilution, ab32072, Abcam, UK) and GAPDH (1:20,000 dilution, Abcam, UK) primary antibodies at 4 °C overnight, followed by another incubation with appropriate HRP-conjugated secondary antibodies for 1 h at room temperature. The protein bands were visualized using a Millipore detection kit (WBKLS0100, Millipore Corporation, USA). The grayscale of protein bands were analyzed using Image J.

### 5-ethynyl-29-deoxyuridine (EdU) assay

Cells were collected by 0.25% trypsin (Gibco, NY, USA) and a density of 2 × 10^5^/ml cells were plated onto 24-well plates (Corning, NY, USA). After 48 h, EdU solution was added to the medium and conducted according to the manufacturer’s instruction (RIBOBIO, China).

### Colony formation assay

Cells were cultured at a density of 2000 cells per well in 6-well plates (Corning, NY, USA) at 37 °C for 7 days. The colonies were stained with Giemsa for 15 min after fix with 4% paraformaldehyde (PFA) (St. Louis, MO, USA) for 30 min.

### Wound scratch assay and transwell assay

Cell migration ability was performed by the wound scratch assay using ibidi Culture Insert Two Wells (ibidi, Germany) following manufacturer’s protocol. Cell migration images were captured at an hourly interval. Transwell assay was examined by chambers precoated with Matrigel (BD, USA). Cells with the density of 10 × 10^4^/ml were suspended to the upper chambers coated with Matrigel. Cells were cultured in at 37 °C for 24 h, invaded cells on the bottom of the chambers were stained with Giemsa and counted in ten random fields with x400.

### Nude mouse xenograft model

Female athymic BALB/c nude mice (5 weeks old) were purchased from GemPharmatech Animal Center (Jiangsu, China) and randomly divided into four groups (*n* = 5) for injection with 5×10^6^ A549-HNRNPK, A549-psin-HA, A549-shHNRNPK and A549-scramble, respectively. Lung cancer cells in 200 μL PBS were injected into the right flank of nude mice. Tumor size was measured and recorded with vernier calipers every 4 days. Four weeks after orthotopic injection, the tumor-bearing mice were sacrificed by cervical dislocation. Then, tumors in each group were harvested, and the weights of tumors were recorded. All animal experiments were approved by the Ethics Committee of the First Affiliated Hospital of Hainan Medical University.

### Immunohistochemistry (IHC)

IHC was performed as previously described^44^. Briefly, tissue samples were fixed in 10% PFA and embedded in paraffin. Sections (4 mm) were treated with 3% hydrogen peroxide and 0.05 mol/l Tris-EDTA solution (PH 9.0) to retrieve antigen after deparaffinization and rehydration. Then incubated with bovine serum albumin (BSA) at 37 ˚C for 1 h. Samples were then either incubated with a rabbit anti-human HNRNPK (1:200 dilution, LSBio, USA), anti-Ki67 (1:200 dilution, Abcam, UK), anti-MYC (1:100 dilution, ab32072, Abcam, UK) or the BSA (as a negative control) at 4 ˚C for overnight. Next, tissue samples were incubated with a goat anti-rabbit secondary antibody (Abcam, Cambridge, UK) for 15 min at 37 ˚C. After washing in PBS, samples were stained with 3,5-diaminobenzidine (DAB) for 2 min. The sections were counterstained with hematoxylin and mounted with neutral gum sealant.

### scRNA-Seq and bulk transcriptome across cancers

Single-cell gene expression profiles across four cancer types were obtained from one of the recent studies^45^. We processed the raw gene expression matrices similar as the original study by Seurat package^46^. First, gene expression matrices of individual sample were merged. Cells with <401 UMIs, <201 expressed genes, >6000 expressed genes or >25% of reads mapping to mitochondrial RNAs were removed. Genes were filtered following the SCENIC. Genes with the total number of reads < 3 UMI count multiplied by 1% of the number of cells and expressed in <1% of cells were removed. The gene expression profiles were normalized and we selected the variable genes based on the same parameters of the original study^45^. The variable genes were used to cluster the cells and clusters were identified by the FindClusters function and visualized by the t-SNE dimensional reduction method^47^. Cells were annotated based on the expression of marker genes. Moreover, we obtained the genome-wide bulk transcriptome and clinical information of lung cancer patients from the The Cancer Genome Atlas (TCGA) project^48^.

### RNA binding proteins and motifs

We obtained the available RBPs and motifs from the ATtRACT database (http://attract.cnic.es)^49^. The position weight matrix (PWM) of RBP motifs were downloaded and transformed to the format of MEME required format^17,50^. There were 3,256 PWMs for 160 RBPs analysed in this study.

### Collection of mRNA sequences

Genome-wide coordinates of protein coding genes were obtained from GENCODE (v35)^51^. Next, the genomic sequences of protein coding genes were downloaded from the UCSC Genome Browser database (https://genome.ucsc.edu)^52^. In total, 19,954 genomic sequences of coding genes were collected.

### Identification of RBP regulators across cell types

To identify the RBP regulators of a cell type of interest, we proposed a computational method RBPreg (Fig. 1A). This method was motivated by the SECNIC pipeline^14^ and there were three main steps in this method. First, we implemented the FIMO algorithm in MEME suite to find the motifs that match in the mRNA sequences. MEME takes advantages of the Expectation Maximization (EM) algorithm to scan the motifs in the sequences^53^. The *p*-value was calculated based on a bootstrap procedure where random sequences with the same length of genes were selected. The RBP motifs with *p* < 1.0E-3 were considered as significant events in the scan. Next, for each motif of RBP, genes were ranked based on the *p*-values. If one mRNA has multiple locations for one motif, we used the most significant one to rank genes. The motifs were classified into two groups based on the affinity between RBP and binding sites. The motifs with significant affinity were considered as directAnnotation, and others were considered as inferredByOrthology.

Moreover, we calculated the spearman correlation coefficient (SCC) between two genes similar as SCENIC and obtained the weight matrices based on GENIE3^16^. The weight represents the RBP has in the prediction of the target. We explored several ways that were also used in SECNIC to determine the threshold and finally obtained the opted targets for each RBP. The first type was taking the 50 genes with highest importance measure (IM) for each RBP (defined as top50). The second one was setting the IM thresholds and IM > 0.001 or IM > 0.005 (defined as w001 and w005). The third type was keeping only the top 5, 10, 50 RBPs for each gene (defined as top5perTarget, top10perTarget, and top50perTarget). In all cases, only the RBP-gene links with IM > 0.001 were considered. Gene sets were split into positive- and negative-correlated targets based on the SCC. Finally, positive gene sets (RBP coexpression modules) with high IMs were remained for further analysis.

Next, we performed the motif enrichment framework and identified the direct targets based on the idea of RcisTarget^54^. We identified the enriched RBP motifs and candidate RBPs for the gene sets identified above. AUCell was used to identify cells with active RBP regulatory networks in single-cell RNA-seq data^14^. The AUC was used to estimate the proportion of genes in the gene-list that were highly expressed in each cell. The cut-offs of AUC score for each gene set in each cell were determined automatically with the ‘AUCell_exploreThresholds’ function. We identified the RBP regulators, in which targets were with significant activities in the cells of interest.

### eCLIP-seq supported RBP-gene regulation

To validate the computationally predicted RBP-gene regulation, we collected the eCLIP-seq data of two cell lines (HepG2 and K562) from the ENCODE project^55^. We downloaded the peak files and mapped the peaks to genes by BEDTools^56^. Genes with peaks were identified as targets of corresponding RBPs. We next calculated the overlap of target genes for RBPs identified from RBPreg and eCLIP-seq supported ones. Hypergeometric test was used to evaluate the significance of the overlap.

### Differential expression analysis

Differential gene expression analysis was performed by DEsingle (V1.10.0)^57^ to identify the genes that were differentially expressed in cell types. The expressions of genes in one cell type vs. all other cell types were compared. Genes with adjusted *p*-values <0.05 were identified and classified into upregulated and downregulated based on the fold changes (FC). Genes with FC > 1 were considered as upregulated.

### Functional analysis of RBPs in cancer

To identify the potential pathways regulated by RBPs, we performed hypergeometric test based on the targets of each RBP prioritized in RBPreg. The cancer hallmark pathways were obtained from MSigDB^58^. Pathways with adjusted *p*-values <0.001 were identified for each RBP regulator. For plotting the circos figure of RBP-gene regulation, only genes annotated in at least two pathways were considered. Moreover, we performed Gene Set Enrichment Analysis (GSEA) for HNRNPK^59^. First, we calculated the SCC between HNRNPK and targets. Next, all target genes were ranked based on SCC and subjected into the GSEA pipeline.

### HNRNPK regulation in cancer

CLIP-seq data of HNRNPK and IgG in HeLa cell line were obtained from Gene Expression Omnibus (GEO) under the accession number GSE127188^60^. Moreover, eCLIP-seq data of HNRNPK in HepG2 and K562 cell lines were obtained from ENCODE^61^. The bigwig files were downloaded and visualized by Integrative Genomics Viewer (IGV)^62^. To further identify the potential targets of HNRNPK, we also downloaded the RNA sequencing data in two cell lines after knockdown HNRNPK. Genes with fold changes > 2 or <0.5 between knockdown HNRNPK and control were identified.

### Classifiers based on expressions of RBPs

To evaluate whether the expressions of RBP regulons can be used for distinguishing different cell types, we constructed the XGBoost classifiers. In each cancer type, the RBPs identified in each cell type were used as features in the classifier. For example, the expressions of RBP regulons identified in T cells were used as features for construction of classifiers for distinguishing T cells from other cell types. In addition, we used the RBPs identified in cancer cells in a specific cancer type to construct the classifiers for distinguishing the cancer cells in the specific cancer type verse other cancer types. The parameters ‘max_depth = 6, eta = 0.5, objective = ‘binary:logistic’, nround = 25’ were used.

### Statistics and reproducibility

All the experiments were set up in triplicate. Statistical analysis was conducted using IBM SPSS Statistics 23.0. The data are expressed as the mean ± SEM. Unpaired, two-tailed Student’s *t*-tests were used for data comparison between two groups. Histograms were performed generated using the GraphPad software. A value of *P* < 0.05 was considered statistically significant.

### Reporting summary

Further information on research design is available in the Nature Portfolio Reporting Summary linked to this article.

## Supplementary information

Supplementary Information

Description of Additional Supplementary Files

Supplementary Data 1

Supplementary Data 2

Supplementary Data 3

Supplementary Data 4

Supplementary Data 5

Supplementary Data 6

Supplementary Data 7

Supplementary Data 8

Supplementary Data 9

Supplementary Data 10

Supplementary Data 11

Supplementary Data 12

Supplementary Data 13

Reporting Summary

## Data Availability

All the data generated in this study can be accessed from Supplementary Data 1–13, source data for Figs. 3c, d, e, 4a, c, d, e, f, and 6g were provided in Supplementary Data 13. The uncropped images of gel(s)/blot(s) in Fig. 6g were provided in Supplementary Fig. S18.

## Code availability

All the codes can be obtained from https://github.com/ComputationalEpigeneticsLab/RBPreg and the web server of BRPreg can be accessed from http://bio-bigdata.hrbmu.edu.cn/RBPreg/.
