# Supplementary material for: Single-cell RNA binding protein regulatory network analyses reveal oncogenic HNRNPK-MYC signalling pathway in cancer
Source: Commun Biol. 2023 Jan 21;6:82. doi: 10.1038/s42003-023-04457-2 (PMC9867709; doi:10.1038/s42003-023-04457-2)
Supplement: Supplementary file 1 — Supplementary Information [file 42003_2023_4457_MOESM1_ESM.pdf]

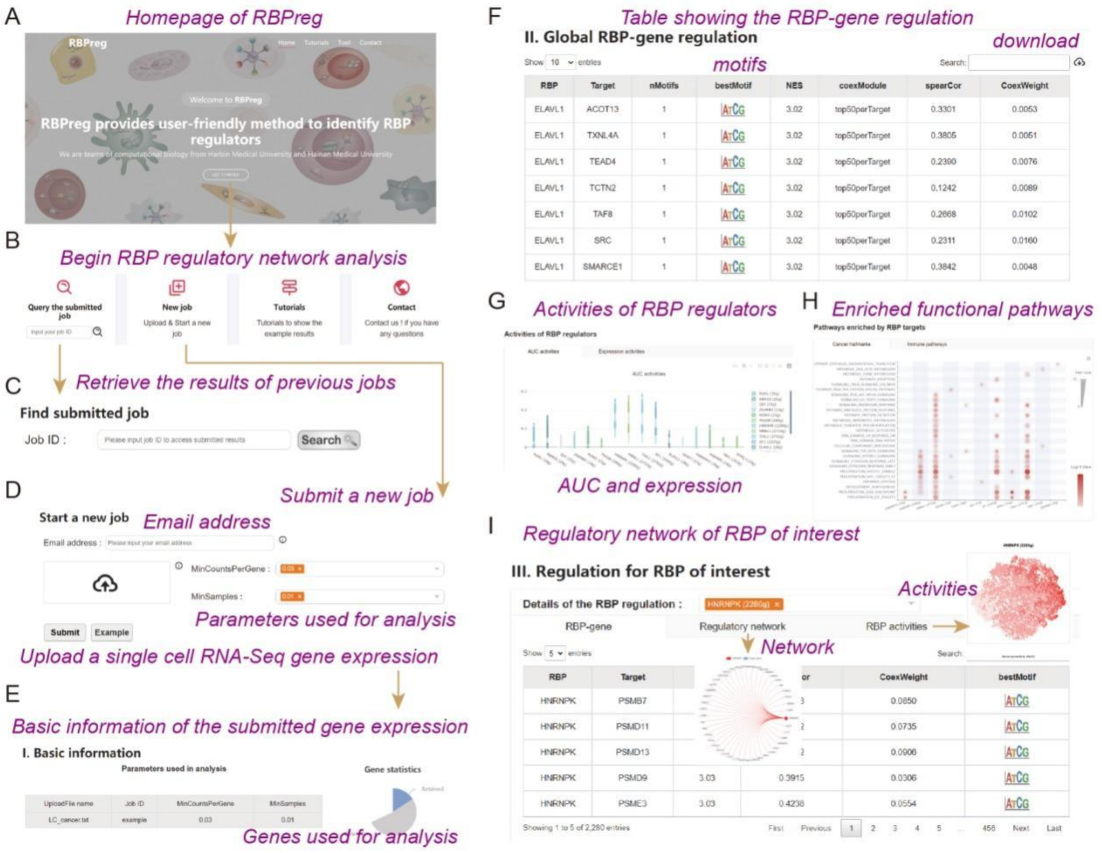

Fig. S1. Workflow for using the RBPreg web server. A, Homepage of RBPreg web server. B, Four functional modules in RBPreg web server. C, Retrieve the results of previous jobs. D, Submission of a new job in RBPreg. E, The basic information of the submitted gene expression profiles. F, Table showing the identified RBP-gene regulation. G, Activities of RBP regulators. H, Enriched functional pathways by potential targets of RBP regulators. I, Network view of RBP-gene regulation.

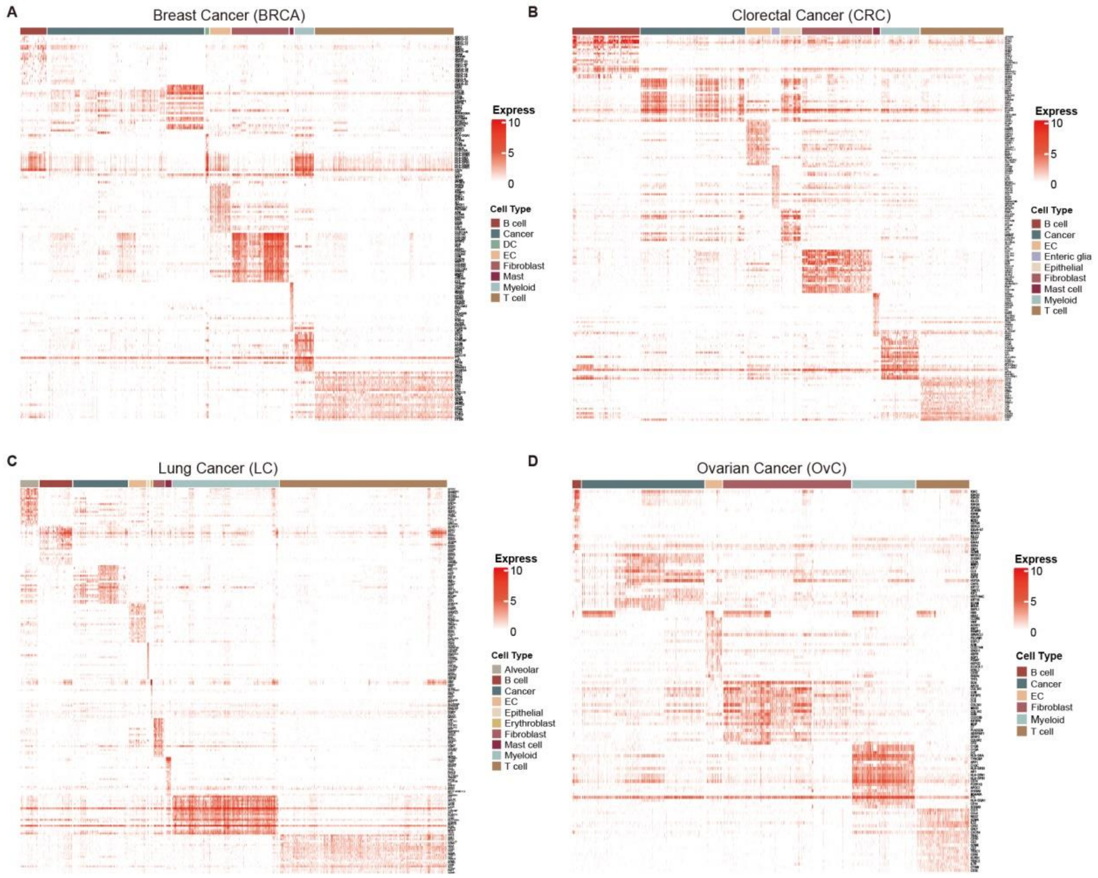

Fig. S2. Heat maps showing the expression of marker genes in different cell types across cancer types. A for BRCA, B for CRC, C for LC and D for OvC.

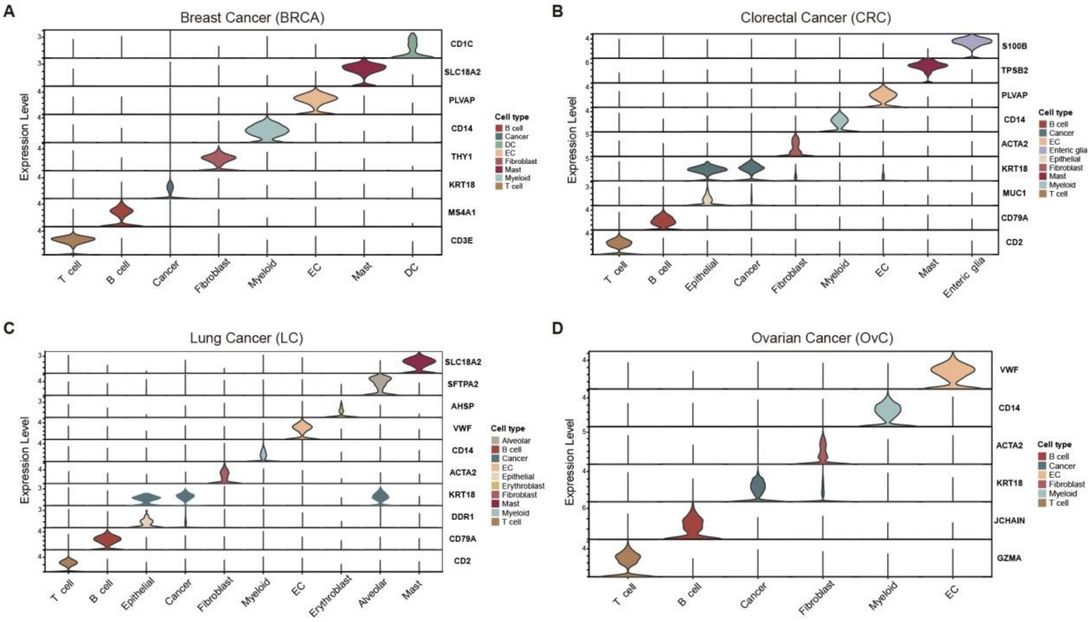

Fig. S3. Violin plots for expressions of marker genes in different cell types across cancer types.

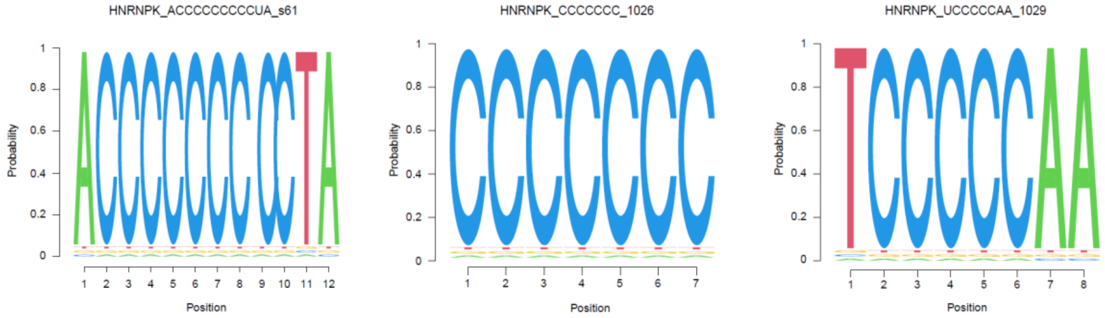

Fig. S4. The significantly enriched motifs for HNRNPK in lung cancer.

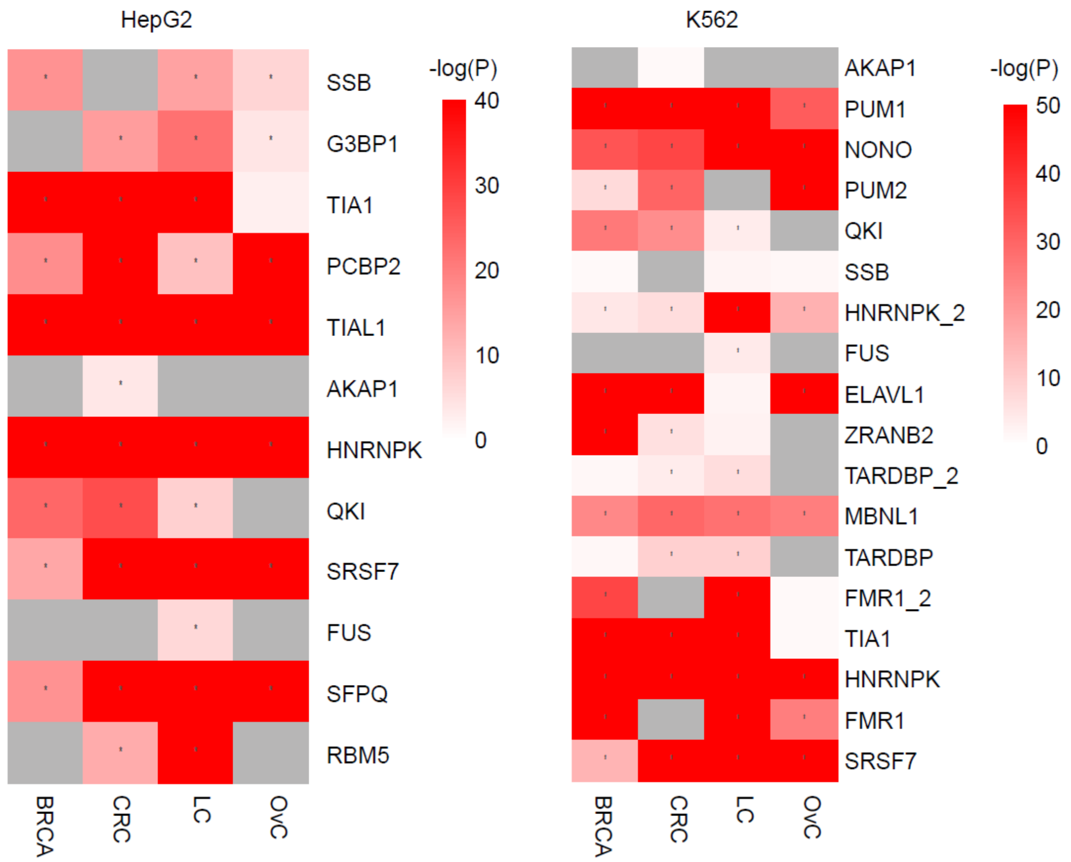

Fig. S5. Overlap of target genes for RBPs between computationally predicted and eCLIP-Seq supported across cancer types. Left for HepG2 cell line and right panel for K562 cell line. *p<0.05.

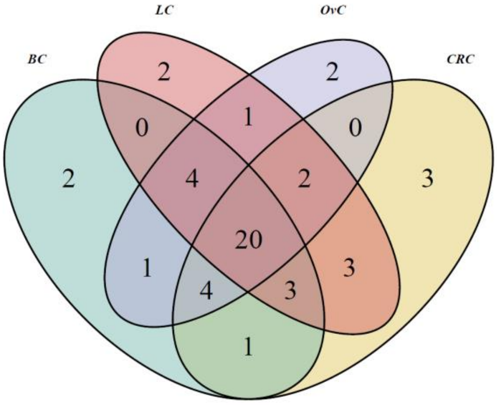

Fig. S6.Venn plot showing the overlap of RBP regulators across four cancer types.

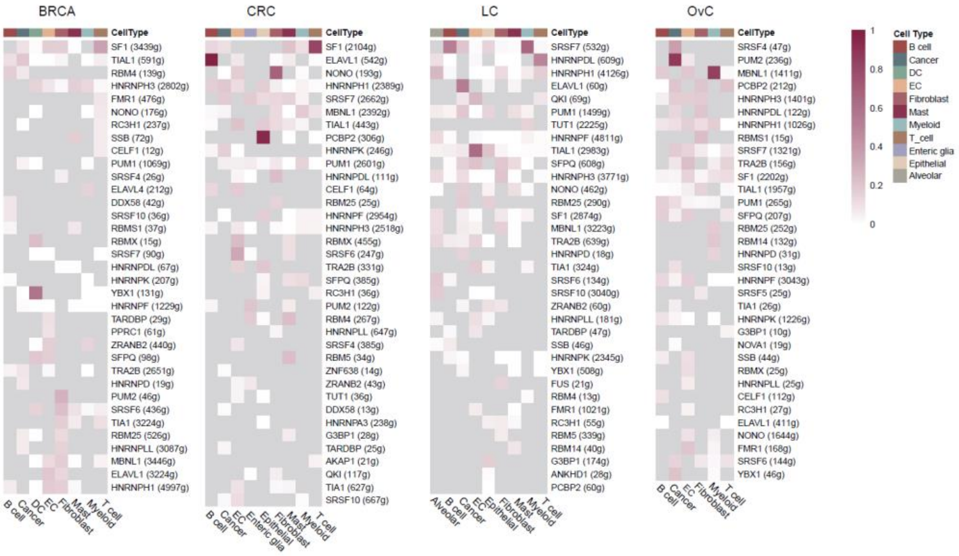

Fig. S7. Heat maps showing the proportions of activated cells in each cell type across cancer types. Gray means that the RBP was not identified in this cell type.

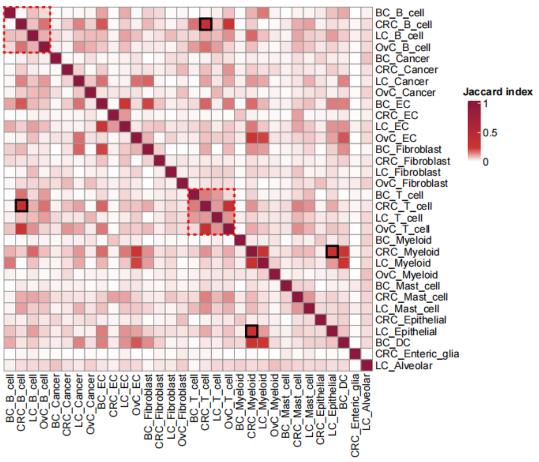

Fig. S8. Overlap of RBP-gene regulatory network across cell types in four cancer types. The panel showing the jaccard index between two networks. T cells and B cells

were labeled with red lines. Comparisons with jaccard index > 0.3 were labeled with dark black lines.

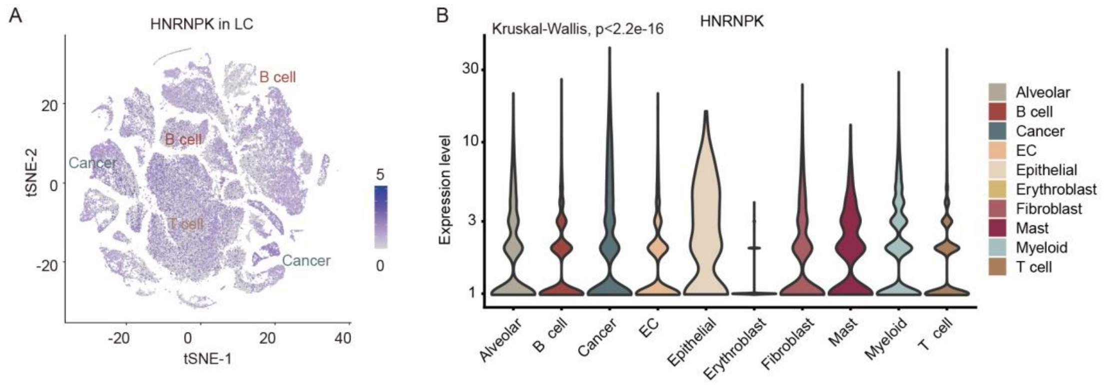

Fig. S9. Expressions of HNRNPK in different cell types of lung cancer. A, t-SNE plot showing the expression of HNRNPK in cells. B, Violin plots showing the expression of HNRNPK across cell types in lung cancer.

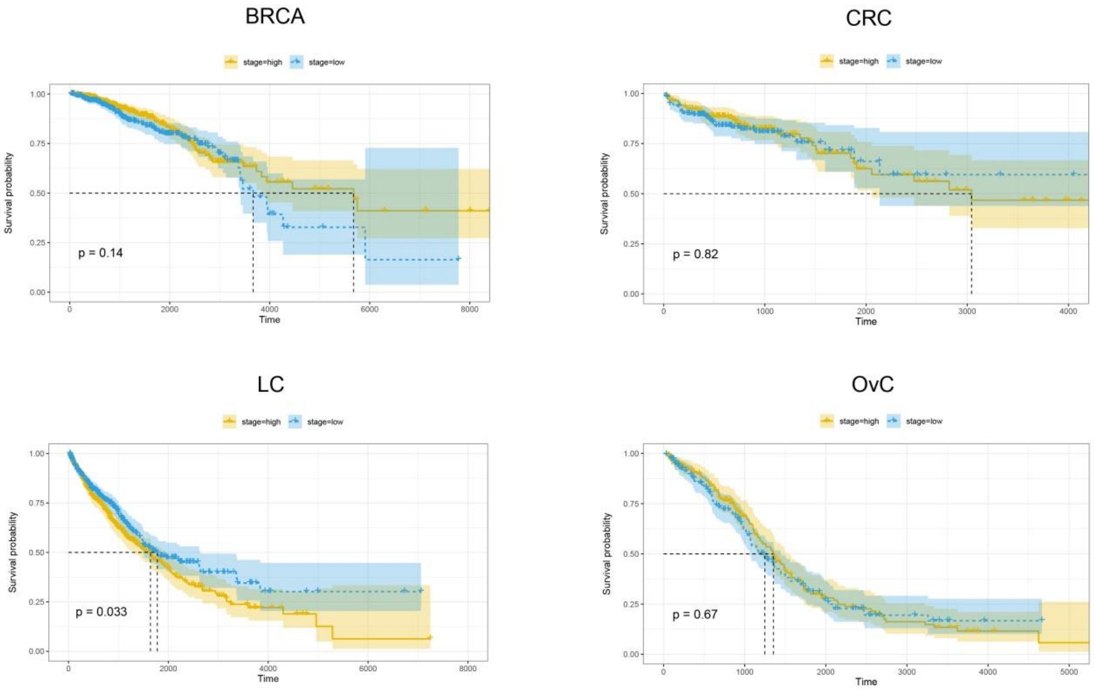

Fig. S10. Association of HNRNPK with survival across cancer type.

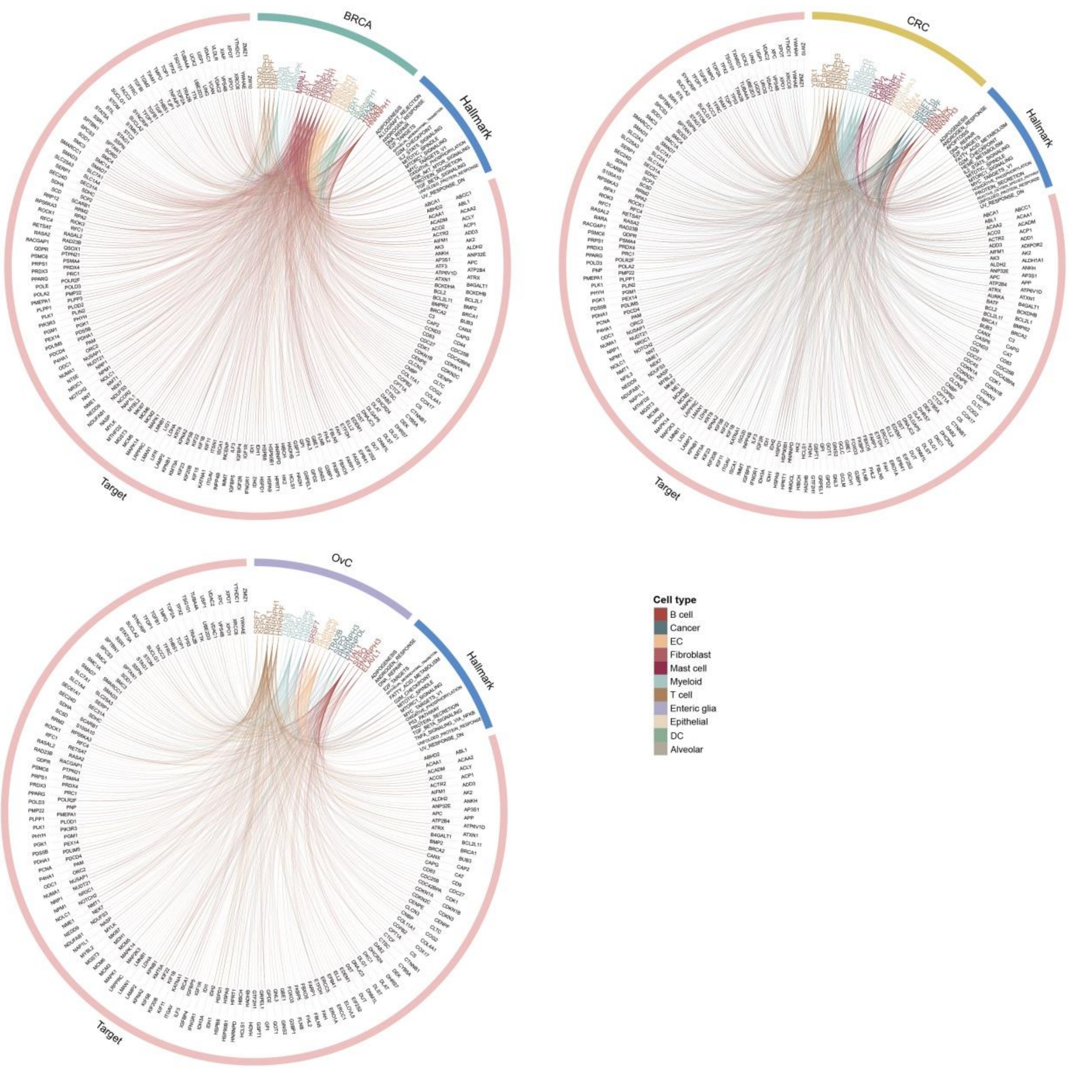

Fig. S11. Circos plots showing the RBP-gene-cancer hallmark associations across cancer types.

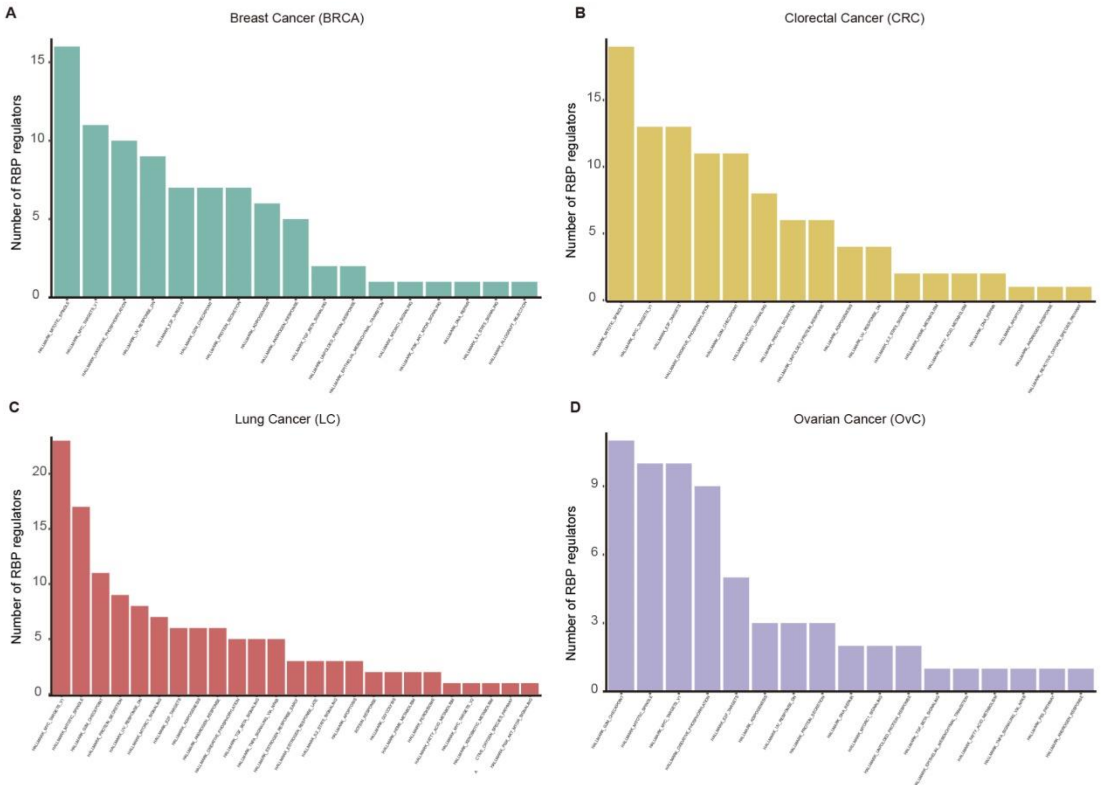

Fig. S12. Numbers of RBP regulators for cancer hallmark pathways across cancer types. A for BRCA, B for CRC, C for LC and D for OvC.

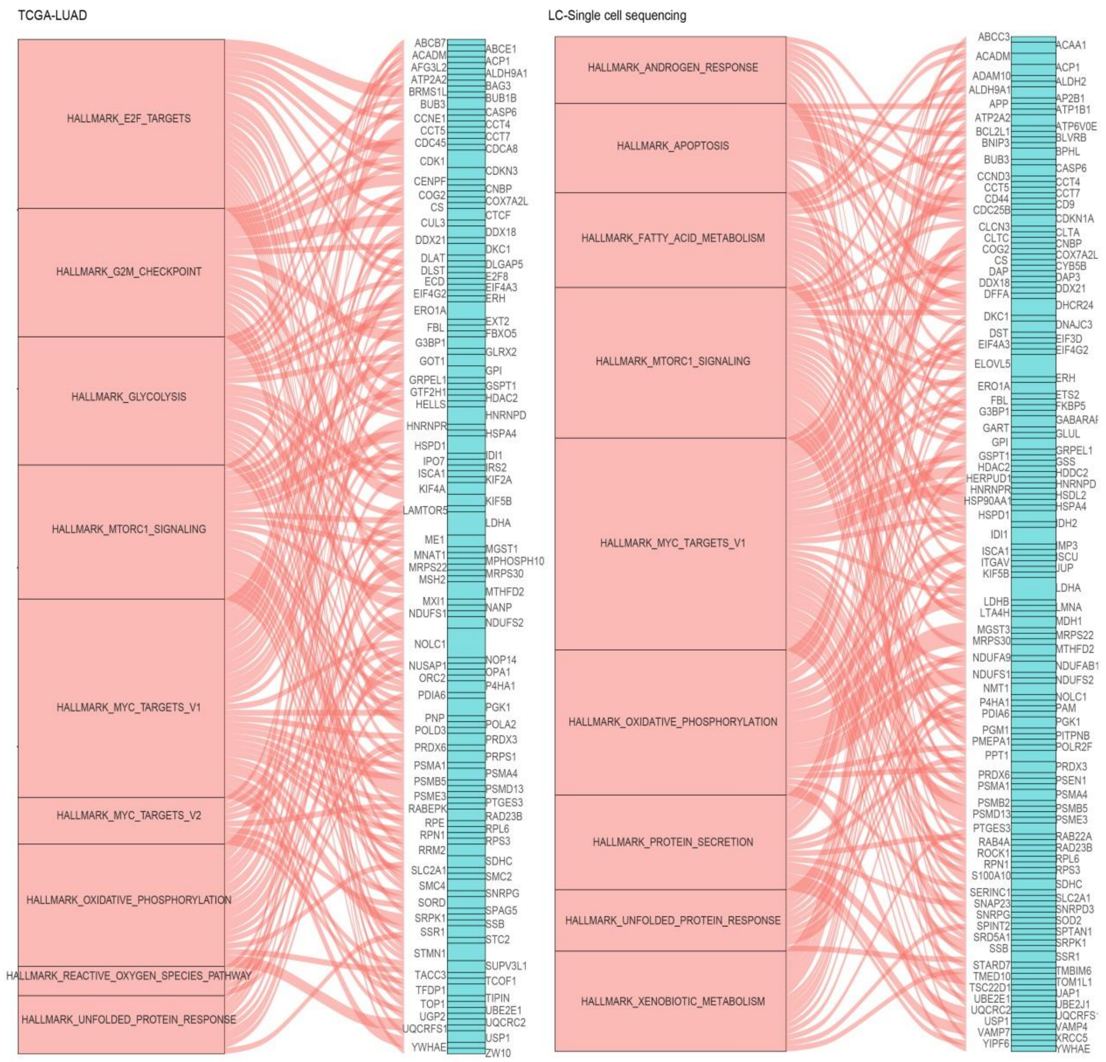

Fig. S13. River plots showing the associated pathways of HNRNPK target genes. Left panel for TCGA-LUAD data and right panel for single cell sequencing data.

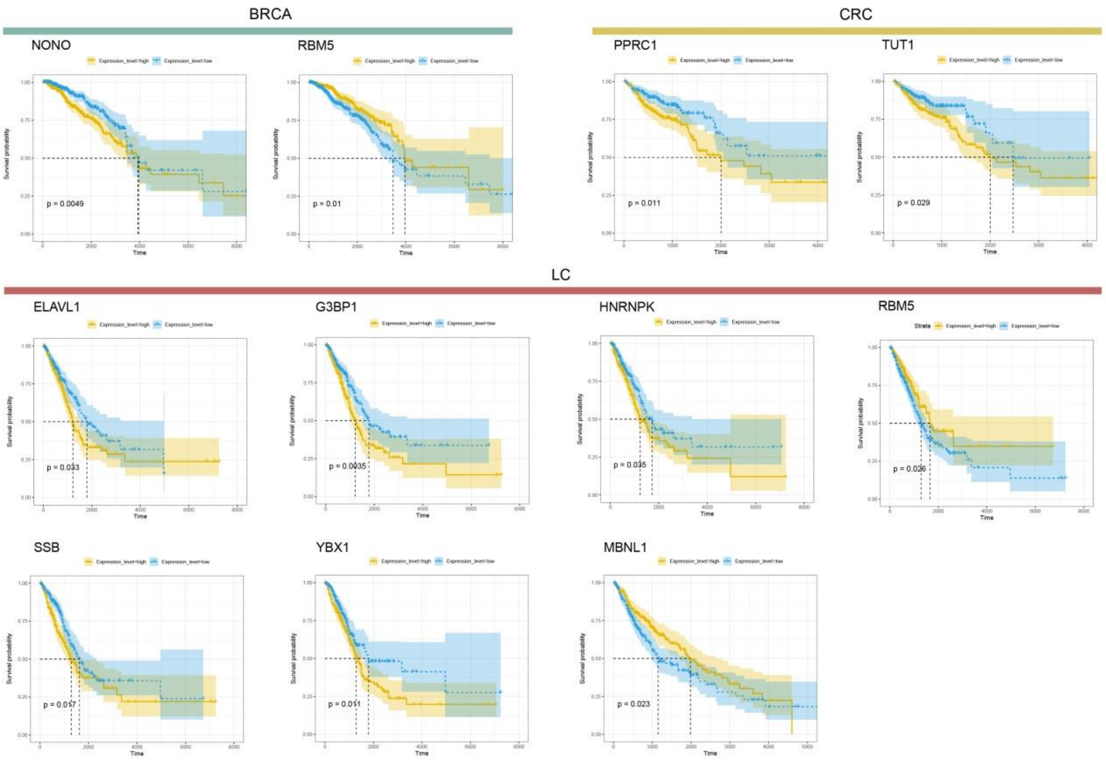

Fig. S14. Kaplan-Meier survival analysis of cancer patients stratified by the median expression levels of RBPs.

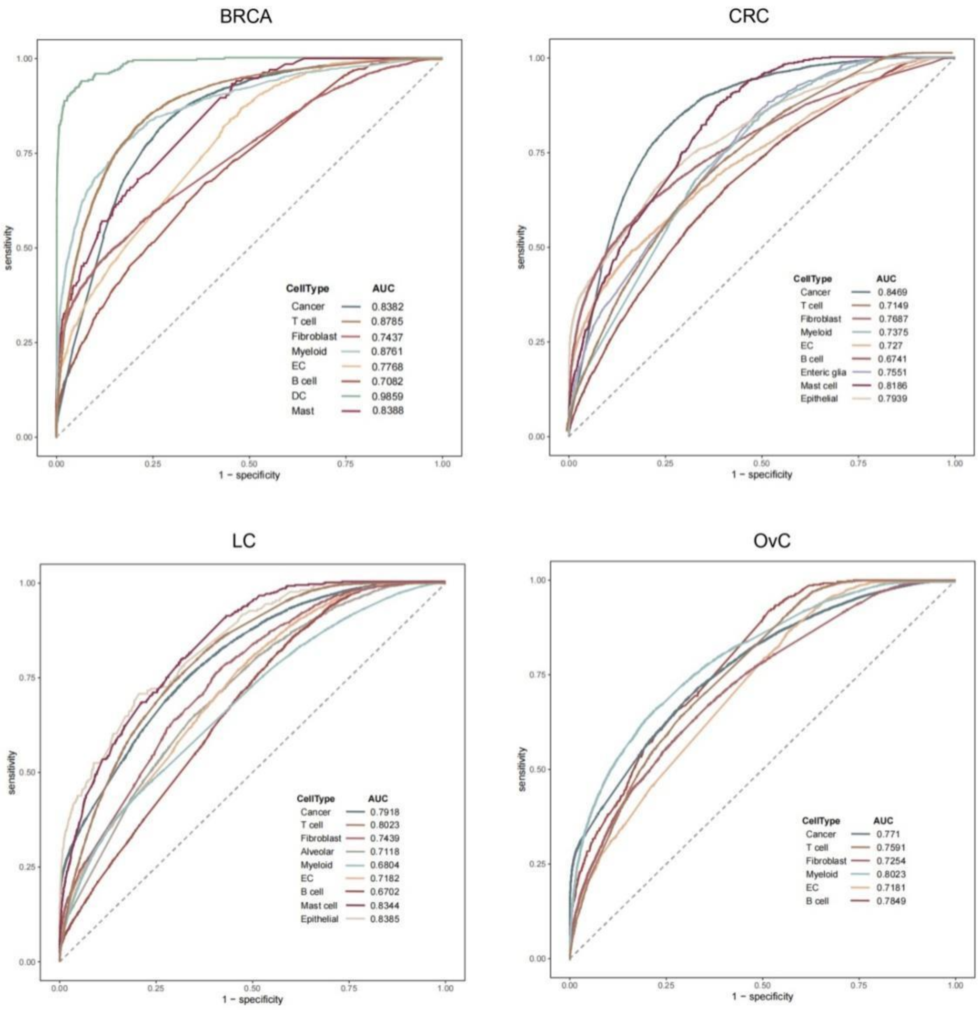

Fig. S15. ROCs of xgboost classifiers based on the expression of RBP regulators.

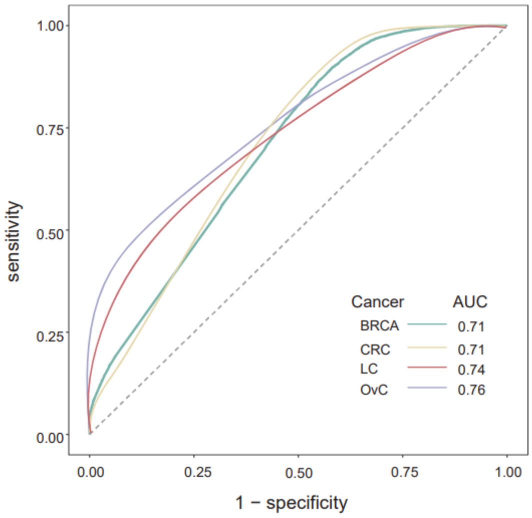

Fig. S16. ROCs of xgboost classifiers based on the expression of RBP regulators for distinguishing cancer cells from different cancer types.

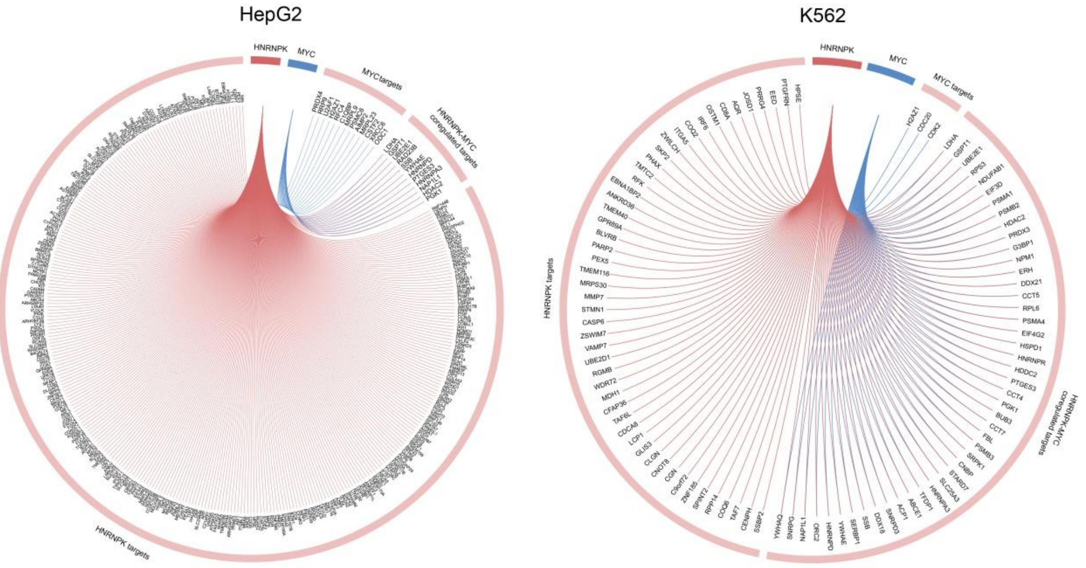

Fig. S17. HNRNPK and MYC regulatory networks in two cell lines. Left for HepG2 and right for K562.

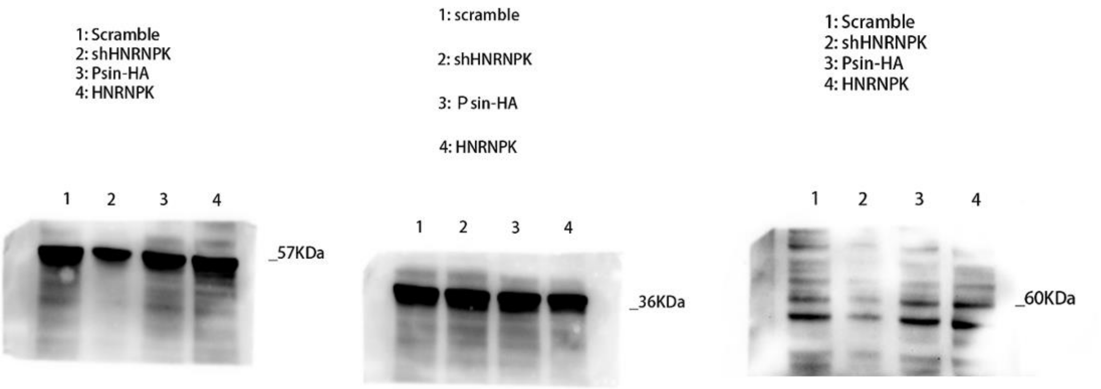

Fig. S18. The uncropped images of gel(s)/blot(s) in Figure 6g.
