# Supplementary material for: Single-cell RNA binding protein regulatory network analyses reveal oncogenic HNRNPK-MYC signalling pathway in cancer
Source: Commun Biol. 2023 Jan 21;6:82. doi: 10.1038/s42003-023-04457-2 (PMC9867709; doi:10.1038/s42003-023-04457-2)
Supplement: Supplementary file 2 — Description of Additional Supplementary Files [file 42003_2023_4457_MOESM2_ESM.pdf]

# Description of Additional Supplementary Files

**File name:** Supplementary Data 1

**Description:** RBP motifs information mapping to exons and introns.

**File name:** Supplementary Data 2

**Description:** The marker genes for cell types across cancers.

**File name:** Supplementary Data 3

**Description:** The annotations of cell types in four cancer types.

**File name:** Supplementary Data 4

**Description:** The RBP regulators in cell types.

**File name:** Supplementary Data 5

**Description:** The enriched RBP motifs in each cancer type.

**File name:** Supplementary Data 6

**Description:** Jaccard index of RBP-gene regulation.

**File name:** Supplementary Data 7

**Description:** Differential expression of RBPs in four cancer types.

**File name:** Supplementary Data 8

**Description:** Literature annotations of RBP regulators.

**File name:** Supplementary Data 9

**Description:** RBP-gene-Cancer hallmark associations across cancer types.

**File name:** Supplementary Data 10

**Description:** Functional enrichment analysis of RBP regulators.

**File name:** Supplementary Data 11

**Description:** Clinical survival associated RBPs.

**File name:** Supplementary Data 12

**Description:** The sequences of primers used in this study.

**File name:** Supplementary Data 13

**Description:** Source data for figure 3, 4 and 6.
